# A simple method for mitochondrial respiration and calcium uptake assessment in pollen tubes

**DOI:** 10.1016/j.mex.2019.07.023

**Published:** 2019-07-25

**Authors:** Diana J. Ortiz-Jiménez, Casandra M. López-Aquino, Cesar Flores-Herrera, Gisela Preciado-Linares, Israel Gonzalez-Vizueth, Roeb García-Arrazola, Daniela Araiza-Olivera, Manuel Gutiérrez-Aguilar

**Affiliations:** a Departamento de Bioquímica, Facultad de Química, Universidad Nacional Autónoma de México, Ciudad Universitaria, 04510, México City, Mexico; b Departamento de Alimentos y Biotecnología, Facultad de Química, UNAM, Circuito Exterior, Ciudad Universitaria 04510. México City, Mexico; c Instituto de Química, Universidad Nacional Autónoma de México, Circuito Exterior, Ciudad Universitaria 04510. México City, Mexico

**Keywords:** Pollen tube mitochondrial respiration and calcium transport, Pollen tube mitochondria, Mitochondrial calcium uptake, Mitochondrial respiration

## Abstract

Key mitochondrial processes are known to be widely conserved throughout the eukaryotic domain. However, the scarce availability of working materials may restrict the assessment of such mitochondrial activities in several working models. Pollen tube mitochondrial studies represent one example of this, where tests have been often restricted due the physical impossibility of performing experiments with isolated mitochondria in enough quantities. Here we detail a method to measure *in situ* mitochondrial respiratory chain activity and calcium transport in tobacco pollen tubes.

• *Digitonin-mediated plasmalemma permeabilization allows efficient assessment of mitochondrial respiration and calcium uptake.*
• *This method allows quick, reliable and portable measurements from low to high cellular densities, versus methods requiring intracellular calcium reporters.*

## Graphical abstract

**Figure:**
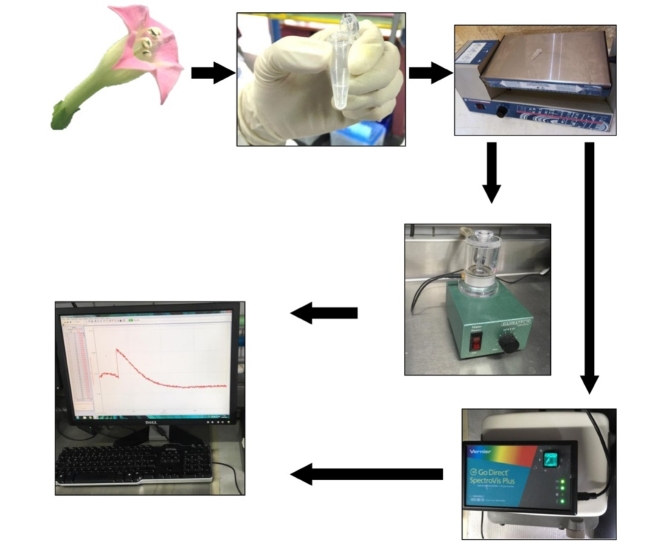

**Specifications Table**

**Table:** 

Subject area:	*Biochemistry, Genetics and Molecular Biology*
More specific subject area:	*Plant mitochondrial physiology*
Method name:	*Pollen tube mitochondrial respiration and calcium transport*
Name and reference of original method:	*C. Flores-Herrera, G. Preciado-Linares, I. Gonzalez-Vizueth, N. Corona de la Peña, M. Gutiérrez-Aguilar. In situ assessment of mitochondrial calcium transport in tobacco pollen tubes. Protoplasma. 2019 Mar;256(2):503-509. doi: 10.1007/s00709-018-1316-z.*
Resource availability:	*NA*

## Method details

### Background

Pollen tubes require active mitochondrial respiration and copious amounts of calcium to achieve efficient growth [1]. However, the role of substrate-specific mitochondrial respiration and calcium transport for plant and pollen tube metabolism has remained less studied. Recent reports have successfully measured Mitochondrial Calcium Uniporter-dependent calcium uptake activity either using electrophysiology of reconstituted MCU or through heterologous expression of MCU in yeast [2,3]. Here we present methodological details to assess *in situ* mitochondrial respiration and calcium uptake in permeabilized pollen tubes [4]. This method could be applied -in principle- to pollen tubes from any plant provided adequate germination and assay buffers are used throughout the process.

### Materials and reagents

*Nicotiana tabacum* ‘Praecox’ plants at floration stage.

MilliQ water

20 mg/mL Digitonin (Promega).

Dimethyl sulfoxide (Sigma)

1% Evans Blue stock (Sigma)

100μM Ruthenium Red (RuR) stock (Sigma)

2 mM Calcium Green-5N stock (Molecular Probes)

10 mM Calcium Chloride stock (Sigma)

0.5 M Sodium Succinate stock (Sigma)

mM Adenosine Diphosphate stock (Sigma)

1 mg/mL Oligomycin stock (Sigma)

200 mM 2-(*N*-morpholino)ethanesulfonic acid stock pH = 5.7

100mM KCN (Sigma)

500 mM Salicylhydroxamic Acid [SHAM] (Sigma)

*Pollen Germination (PG) Buffer:*

10% sucrose

200μM CaCl_2_

100μM Ca(NO_3_)_2_

1.6 mM H_3_BO_3_

15 mM MES pH = 5.7

*Pollen Tube Assay (PTA) Buffer:*

330mM Mannitol

1 mM KH_2_PO_4_

0.1 mM EDTA

10 mM MES pH 5.7

*Important: Filter sterilize both buffers and store at room temperature.*

*Equipment*

Optical Microscope

Centrifuge for Eppendorf tubes

SpectroVis Plus Bluetooth© enabled mini fluorimeter

Magnetic stirrer at 60 Hz

Micro magnet for cuvette

Hematocytometer (Neubauer chamber)

Micropipettors

1.5 mL Eppendorf tubes

Liquid-Phase Hansatech Oxygraph Plus System (Hansatech Instruments)

Orbital shaker

Temperature-controlled incubator

## Assay procedure

*Pollen harvest*

*1 Fill an Eppendorf tube with 1.0 mL PG buffer.*

*2 Select recently flourished flowers and peel of petals with the aid of scissors and tweezers.*

*3 Dip anthers into the Eppendorf tube containing PG buffer.*

*4 Release pollen grains into PG buffer by mildly shaking the anthers.*

*Pollen tube germination*

*5 Close the Eppendorf tube and gently stir at 60 rpm in an orbital shaker at 28 °C for* 2 h.

*6 Place 20*μL of the suspension into the hematocytometer sample port and determine cell count by multiplying by the chamber predetermined factor.

*7* Wash pollen tubes twice in 1 mL PTA buffer by spinning at 3000 rpm for 20 s.

*Oxygen consumption assay*

*9* Calibrate the Liquid-Phase Hansatech Oxygraph Plus System according to the manufacturer instructions.

*10* Resuspend pollen tubes in a final volume of 0.5 mL containing 1 × 10^6^ pollen tubes into the Oxygraph´s water-jacketed chamber containing PTA buffer with 10 mM sodium succinate, 0.01% digitonin and 200 μM ADP.

*11* Close the chamber with the included plug and make sure no air bubbles remain inside.

*12* Allow five minutes for efficient pollen tube permeabilization and baseline obtention.

*13* Measure oxygen consumption rate for 1–2 min (state 3).

*14* Inject 2 μg oligomycin and measure oxygen consumption rate for 1–2 min (state 4).

*15* Calculate the Respiratory Control Ratio (RCR) by dividing State 3 by State 4.

*16* Add 100 μM KCN plus 500 μM SHAM to inhibit mitochondrial respiration.

*Calcium transport assay*

*17* Resuspend pollen tubes in PTA buffer containing 10 mM sodium succinate, 0.01% digitonin, 20 μM EDTA and 2 μM Calcium Green-5N.

*18* Place pollen tube suspension in a cuvette inside the fluorimeter with mild stirring at 60 rpm. IMPORTANT: Make sure the cuvette port is located at the center of the magnetic stirrer to ensure constant and laminar (non-turbulent) stirring.

*19* Turn on and connect the mini fluorimeter either though Bluetooth® or with a USB cable.

*20* Open Logger Pro and select ‘500 nm fluorescence mode’ under the sensor SpectroVis plus configuration icon.

*21* Select the 530 nm acquisition box and ‘Absorbance versus time’ under the spectrophotometer configuration icon. IMPORTANT: Make sure data collection is set to 1 sample/second and select the appropriate acquisition time intervals for each experiment. A value of 2000s is a good start.

*22* Start recording baseline fluorescence for 500 s.

*23* Add desired amount of calcium (a pulse around 50 μM is usually a good starting point).

*24* Measure calcium transport for at least 2000s and save data.

*25* Start a new trace and measure calcium transport in the presence of freshly made 100 nM ruthenium red (RuR) as negative control. Important: A 100 μM RuR stock solution must be freshly made.

*26* Perform required biological replicates and quantify calcium transport rates (fold changes) in arbitrary units. This can be easily done under Vernier’s Logger Pro® or Excel®.

## Method validation

**Fig. 1:**
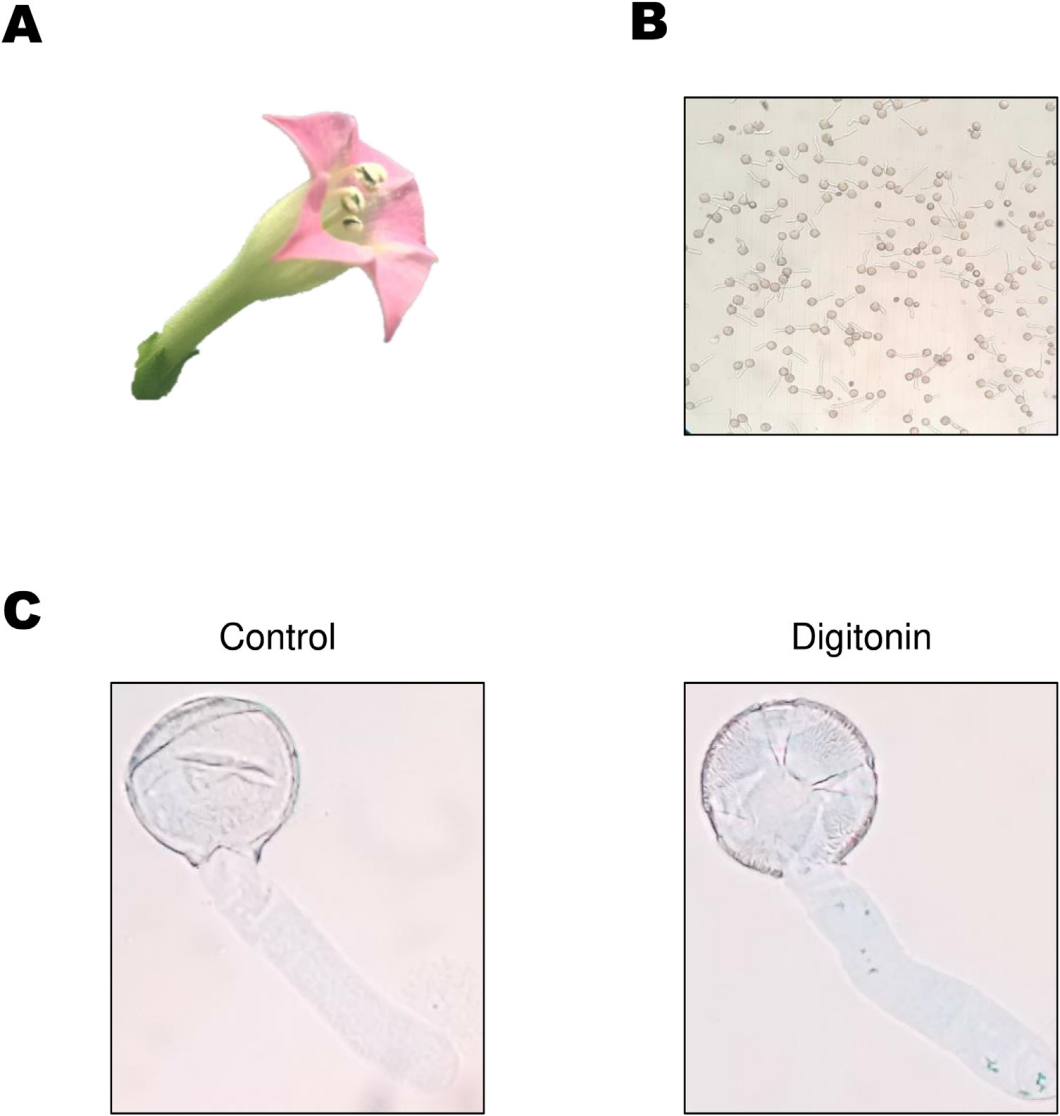
Pollen tube germination (A,B) and plasmalemma permeabilization assessment (C). Pollen tubes were allowed to grow for 2 h, the cells were counted and permeabilized with 0.01% digitonin or 0.5% DMSO. Plasma membrane permeabilization was then assessed by staining with 0.1% Evans Blue. Representative experiments n = 4.

**Fig. 2:**
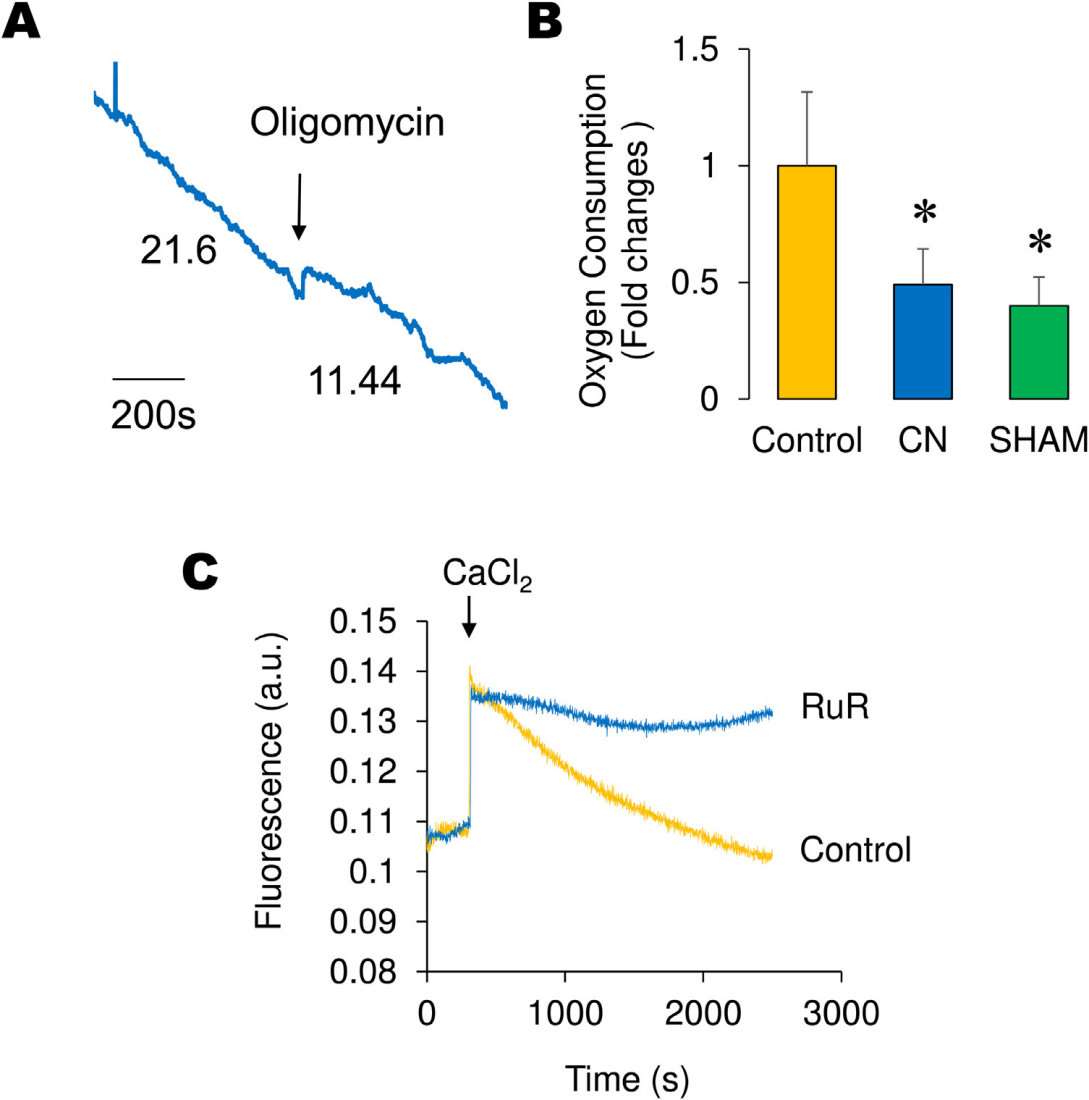
*In situ* monitoring of oligomycin-sensitive respiration (A), CN- and SHAM-sensitive respiration (B) and RuR-sensitive calcium transport activity in permeabilized pollen tubes (C). Permeabilized pollen tubes were resuspended inside the oxygraph’s reaction chamber and state 3 respiration was measured. State 4 respiration was then assessed by adding 2 μg oligomycin (A). Respiration in the absence or presence of cytochrome oxidase inhibitor (cyanide) or alternative oxidase inhibitor (SHAM) was then assessed (B). Data are presented as mean + S.E.M. Statistical evaluation between groups was performed by unpaired t-tests and a P value < 0.05 was considered as criteria of statistical significance against control conditions, as denoted with an asterisk. In (C) calcium transport was assessed either in the absence (control) or presence of 100 nM freshly made RuR. Representative experiments n = 4.

In order to test the method, pollen grains were isolated from mature flowers (Fig. 1A) hydrated in PG buffer and incubated for 2 h. As expected, pollen tubes emerged and were counted in the Neubauer chamber (Fig. 1B). Cell number was determined and adjusted either for oxygen consumption experiments or calcium transport assessment. Samples were then washed twice in PTA buffer in order to remove excess calcium. Pollen tubes were then permeabilized with 0.01% digitonin or 0.5% DMSO (control) for 5 min and stained with 0.1% Evans Blue solution. As expected, permeabilized pollen tubes were stained with Evans Blue (Fig. 1C). In parallel, a separate batch of pollen tubes were treated with 0.01% digitonin for five minutes inside the oxygraph´s chamber and ADP was added to obtain state 3 respiration (Fig. 2). Oligomycin addition resulted in a transient decrease in respiration typically associated with ADP phosphorylation blockade (Fig. 2A). Furthermore, addition of 100 μM KCN significantly decreased state 3 respiration whereas subsequent addition of 500 μM SHAM resulted in no significant changes when compared with respiration in the presence of cyanide (Fig. 2B).

Once mitochondrial coupling was determined, mitochondrial calcium uptake was assessed by placing pollen tubes in a mini fluorimeter with constant stirring in the presence of 10 mM succinate and 2μM calcium green. A single pulse of 100μM CaCl_2_ was then added and fluorescence spiked to a maximum value followed by a constant fluorescence decrease indicating calcium transport (Fig. 2C). Under the same conditions, preincubation with 100 nM RuR significantly inhibited such fluorescence decrease, indicating calcium transport blockade. These results indicate the in-situ method is also suitable for assessing mitochondrial respiration and calcium transport in permeabilized pollen tubes.

## Funding

This work was supported by a grant from Dirección General de Asuntos del Personal Académico, Programa de Apoyo a Proyectos de Investigación e Innovación Tecnológica (PAPIIT)IA203419 and UNAM-FQ-PAIP 5000-9171 (to M.G-A.).
